# Edge Intelligence-Driven Bearing Fault Diagnosis: A Lightweight Anti-Noise Diagnostic Framework

**DOI:** 10.3390/s26072063

**Published:** 2026-03-26

**Authors:** Xin Lin, Wei Wang, Xinping Peng, Bo Zhang, Lei Liu

**Affiliations:** 1 Equipment and Technology Center of the National Railway Administration, Beijing 100070, China; 2 State Key Laboratory of Heavy-Duty and Express High-Power Electric Locomotive, Zhuzhou 412001, China; 3 CRRC Zhuzhou Locomotive Co., Ltd., Zhuzhou 412001, China; 4 State Key Laboratory of Rail Transit Vehicle System, Southwest Jiaotong University, Zhuzhou 412001, China

**Keywords:** anti-noise, bearings, dynamic input, edge intelligence, fault diagnosis, lightweight network

## Abstract

Edge intelligence enables significant latency reduction and enhances the timeliness of model-based fault diagnosis. However, existing deep learning-driven bearing fault diagnosis models are ill-suited for deployment on edge devices, primarily due to three critical limitations: (1) Lightweight models typically exhibit inadequate anti-noise performance, failing to meet the reliability requirements of real-world engineering scenarios. (2) Models with superior anti-noise capabilities often demand high-performance hardware for operation, thereby restricting their deployment on resource-constrained edge devices. (3) These models adopt a fixed input length, which makes it difficult to guarantee diagnostic accuracy across diverse application scenarios—attributed to variations in sampling frequencies, bearing parameters, and other relevant factors. To address these challenges, this paper proposes a lightweight anti-noise diagnostic framework (LADF) for edge-intelligent bearing fault diagnosis in complex engineering environments. The LADF comprises three core modules: a dynamic input module (DIM), a lightweight network module (LNM), and a denoising branch. Specifically, the DIM is designed based on the envelope spectrum, leveraging its inherent demodulation characteristics to dynamically adapt to input signals across diverse scenarios. Group convolution and layer normalization are employed to construct the LNM, ensuring robust diagnostic performance while achieving efficient computation. The denoising branch constrains the feature extractor via a loss function, enabling it to learn generalized fault features under varying noise environments and thereby enhancing the anti-noise capability of the framework. Finally, the proposed LADF is validated through test rig experiments on two datasets of train axle box bearings. Comparative analysis with state-of-the-art models demonstrates that the LADF achieves superior diagnostic stability and anti-noise performance while maintaining a more lightweight architecture, making it well-suited for edge deployment in railway bearing fault diagnosis.

## 1. Introduction

Rail transportation underpins modern industrial society and is instrumental in driving socioeconomic progress [1,2,3]. As a critical component of the bogie system, Railway Axle Box Bearings (RABB) bear the brunt of extremely complex operational conditions marked by highly variable environments and alternating loads. These harsh factors predispose RABBs to frequent damage. Any failure can result in costly operational downtime, disrupt train scheduling, and even trigger catastrophic accidents jeopardizing passenger safety [4]. Consequently, developing and deploying timely and accurate fault diagnosis models for RABB is of critical importance.

Generally, bearing fault diagnosis approaches can be categorized into three main groups: traditional methods based on signal processing techniques [5], classical machine learning-based methods [6,7], and deep learning-based methods [8,9,10,11,12,13,14]. Traditional approaches typically rely on manual visual inspection, which proves inefficient in the context of big data applications [15]. Likewise, classical machine learning methods depend heavily on manual feature extraction, where model performance is directly influenced by the quality of the selected features. This process demands substantial domain expertise, thereby limiting accessibility for non-specialists [16]. In contrast, deep learning methods have gained widespread popularity due to their ability to automatically learn latent features from raw data, thereby eliminating the need for complex and time-consuming manual feature engineering [17,18,19].

Hardware advancements and the swift expansion of the Internet of Things (IoT) have driven deep learning to deliver impressive results in a wide range of industries [20,21,22]. Nevertheless, the high performance of deep learning models generally relies on powerful computing hardware. In conventional practice, such models are deployed in the cloud, necessitating that edge devices send data for remote processing. This architecture frequently results in inference delays, primarily due to limitations in channel bandwidth.

In response to this challenge, the concept of edge intelligence has gained prominence [23]. It proposes the direct deployment of deep learning models on the edge devices themselves—where data is generated—thereby minimizing communication latency and enabling truly real-time processing. However, the inherently limited resources of edge devices present a fundamental constraint. Consequently, the pursuit of practical edge deployment critically depends on minimizing two key computational burdens: Multiply-Accumulate Operations (MACs) and Memory Footprint (MF). This, in turn, underpins the necessity for dedicated research into lightweight deep learning architectures.

The development of deep learning has evolved from an initial focus on maximizing diagnostic accuracy [24] to a growing emphasis on model efficiency, necessitating lightweight architectures for embedded deployment. Convolutional Neural Networks (CNNs), owing to their weight-sharing property, have become the structural backbone for many lightweight network designs [25,26,27,28,29]. In the field of bearing fault diagnosis, numerous studies have employed 1D-CNNs to construct lightweight models [30,31]. However, these models often exhibit limited robustness against noise interference. Conversely, a range of models with enhanced anti-noise capabilities have been proposed [32,33]; yet, their performance paradoxically suffers a significant decline when confronted with the extreme and complex noise conditions characteristic of real-world RABB data. Furthermore, achieving strong noise robustness frequently comes at the expense of model efficiency. Techniques such as Training Interference CNN [34], Multibranch Multiscale CNN [35], and Feature Attention mechanisms [36] have improved noise tolerance, but often incur substantial computational overhead or lack adaptability to varying and unknown noise intensities.

The current research landscape reveals a notable gap: lightweight diagnostic models often demonstrate insufficient robustness against noise interference, while models designed for enhanced noise robustness seldom maintain a lightweight architecture. Furthermore, a common constraint shared by both types of approaches is their dependence on a fixed input length (e.g., 2048 sample points) [34,37]. This fixed-length requirement hinders the model’s ability to ensure consistent diagnostic accuracy across varying operational scenarios characterized by different sampling frequencies and bearing parameters. Specifically, under high-frequency sampling conditions, a predetermined, fixed input length may inadequately capture complete periodic fault signatures, thereby compromising diagnostic reliability.

In summary, existing models for edge-based RABB diagnosis face three main challenges:

(1) Poor Noise Immunity: lightweight models often lack the anti-noise performance required for real engineering environments.
(2) High Computational Cost: models with robust anti-noise capabilities require hardware resources that exceed the limits of edge devices.
(3) Inflexible Input Lengths: fixed input lengths cannot accommodate varying sampling frequencies and bearing parameters, leading to unstable diagnostic accuracy across different application scenarios.

To address these challenges, this paper introduces a Lightweight Anti-noise Diagnostic Framework (LADF) tailored for edge intelligence applications in RABB fault diagnosis. The proposed LADF integrates three core modules: a Dynamic Input Module (DIM), a Lightweight Network Module (LNM), and a dedicated denoising branch. Specifically, the DIM is designed based on envelope spectrum analysis, exploiting its intrinsic demodulation properties to dynamically adjust to input signals under varying operational conditions. The LNM is constructed using group convolution and layer normalization techniques to maintain diagnostic robustness while optimizing computational efficiency. Furthermore, the denoising branch imposes constraints on the feature extractor through a specialized loss function, enabling it to learn generalized fault representations across diverse noise environments, thereby significantly enhancing the overall noise resilience of the framework.

Experimental validation on two types of RABB datasets demonstrates that the LADF outperforms the state-of-the-art models in terms of stability and noise immunity while remaining computationally lighter. The specific contributions of this paper are as follows:

(1) An LNM is constructed to facilitate efficient edge deployment. By employing group convolution and layer normalization, the LNM significantly reduces the computational cost and model complexity without compromising diagnostic performance, making it highly suitable for resource-constrained edge intelligence applications.
(2) A DIM based on the envelope spectrum is proposed to enhance scenario adaptability. Leveraging the inherent demodulation characteristics of the envelope spectrum, this module enables the framework to dynamically adapt its input signals to diverse operating scenarios, overcoming the limitations associated with fixed-input models.
(3) An auxiliary denoising branch is introduced to strengthen the model’s anti-noise capability. By constraining the feature extractor through a specific loss function, this branch guides the network to learn generalized fault features even under varying noise environments, thereby ensuring robust fault diagnosis in harsh engineering conditions.

The remainder of this paper is organized as follows: Section 2 and Section 3 introduce the theoretical background and the proposed model, respectively. Section 4 details the experiments on two RABB datasets. Section 5 provides a discussion of the results, and Section 6 concludes this paper.

## 2. Theoretical Background

Before elaborating on the structure and working mechanism of the proposed LNM in detail, this section first introduces the two fundamental sub-modules that constitute LNM, namely Group Convolution (GC) and Layer Normalization (LN). As the key components underpinning LNM’s core functions of feature extraction optimization and data distribution regularization, the rationality of their design and the efficacy of their collaborative operation directly determine the feature enhancement performance of LNM in noise-interfered scenarios. It is therefore necessary to clarify the core principles and application value of these two sub-modules first. Upon completing the introduction to the basic sub-modules of the LNM, this section further presents a concise illustration of Envelope Spectrum (ES), the core component of the DIM. As a classical and effective analytical tool for fault feature extraction of vibration signals, ES has been widely applied in the field of RABB fault diagnosis. It can effectively highlight the periodic characteristics of fault impact signals, serving as the core basis for DIM to achieve dynamic fusion of multi-source features and accurate fault identification. Thus, it is essential to clarify its mechanism of action and integration logic within the DIM module of this study.

### 2.1. Group Convolution

**Figure 1:**
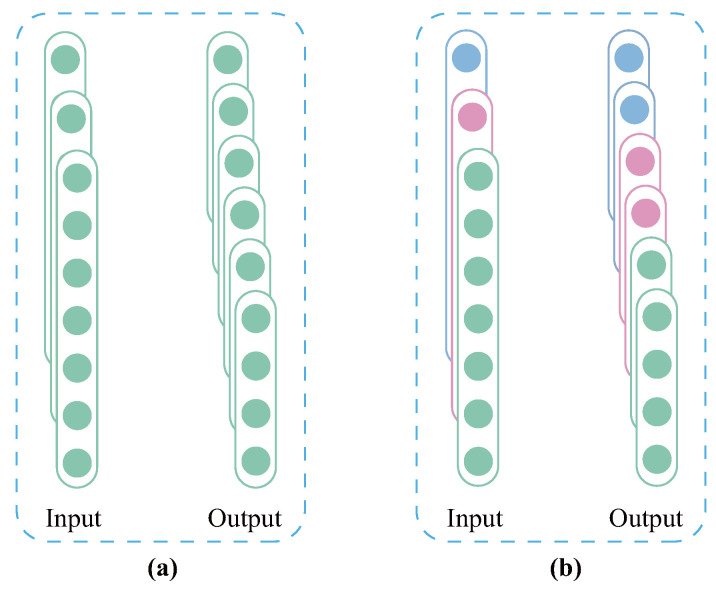
Illustration the difference in convolutional connection methods: (**a**) NC, (**b**) GC (fully connected only if the input and output layers with the same color).

The special connection between the input and output layers of the GC makes it suitable for implementing lightweight network. Normal convolution (NC) as the most used structure in the existing studies [38,39], as shown in Figure 1a, is used as the comparison object to elaborate the lightweight process of the GC. The input channels, output channels, length of the convolution kernel of NC, and length of the feature maps are assumed to be $C_{in}$, $C_{out}$, $L_{k}$, and $L_{f}$, respectively. Then, the number of parameters and MACs of NC are as follows (when stride and padding are 1 and 0, respectively):

(1) $$P_{NC}=\left(C_{in}L_{k}+1\right)C_{out}$$

(2) $${\mathrm{MACs}}_{NC}=C_{in}C_{out}L_{k}(L_{f}-L_{k}+1)$$

Compared with NC, the GC only performs local connection during convolutional operation, as shown in Figure 1b. Then, the number of parameters and MACs of GC can be expressed as follows:

(3) $$P_{GC}=\left(\frac{C_{in}}{G}L_{k}+1\right)C_{out}$$

(4) $$MACs_{GC}=\frac{C_{in}}{G}C_{out}L_{k}(L_{f}-L_{k}+1)$$

where *G* denotes the number of groups into which the convolutional kernels are divided in GC.

By systematically comparing the parameter calculation model of NC described in Equation (1) with the parameter quantification formula of GC corresponding to Equation (3) while integrating the MACs calculation method of the NC architecture in Equation (2) and the MACs derivation expression of the GC architecture in Equation (4), a conclusion with critical guiding significance can be drawn: under the conditions of identical feature map dimensions and convolution kernel configurations, the total parameter volume and MACs of the GC architecture are reduced by nearly *G* times compared with those of the traditional NC architecture. This remarkable dimensionality reduction effect directly endows the GC architecture with prominent lightweight advantages. Therefore, in contrast to the NC architecture with high structural redundancy, GC can more efficiently adapt to the design requirements and engineering deployment scenarios of lightweight networks, providing core technical support for the practical application of models on resource-constrained devices.

### 2.2. Layer Normalization

In the training process of deep learning models, normalization techniques effectively mitigate issues such as gradient vanishing or gradient explosion by standardizing the input features of each network layer, thereby significantly accelerating model convergence. Simultaneously, this technique reduces the impact of feature distribution shifts on model training, enhances the model’s adaptability to different data distribution scenarios, and consequently improves its generalization performance. Owing to these core advantages, normalization has become an indispensable and critical component in the design of deep learning model architectures and is widely applied across various fields, including image recognition, natural language processing, and industrial fault diagnosis.

Currently, a variety of normalization methods are commonly used in the field of deep learning, each with characteristics suited to different network structures and task scenarios. These mainly include Batch Normalization (BN) [40], Group Normalization (GN) [41], Instance Normalization (IN) [42], and LN. Among them, BN is particularly widely applied in the field of bearing fault diagnosis due to its effectiveness in handling feature distribution fluctuations during training with mini-batches and its significant improvement in training stability for deep convolutional neural networks—bearing fault diagnosis data often suffers from issues such as uneven sample distribution and significant environmental noise interference. BN enhances the model’s ability to extract fault features and improves diagnostic accuracy and reliability by standardizing features across batch samples.

To lay the groundwork for the subsequent derivation and explanation of the mathematical expression for LN, we first clarify the core parameter definitions of the feature map. Assume the feature map input to the normalization layer, with its batch size (BS), channel number (C), and feature map length (L) defined as *B*, *C*, and *L*, respectively. Here, batch size *B* represents the number of samples input in a single training iteration, channel number *C* corresponds to different dimensions of feature information in the feature map, and length *L* denotes the sequence length of features on a single channel. Together, these three parameters define the three-dimensional tensor structure of the feature map.

**Figure 2:**
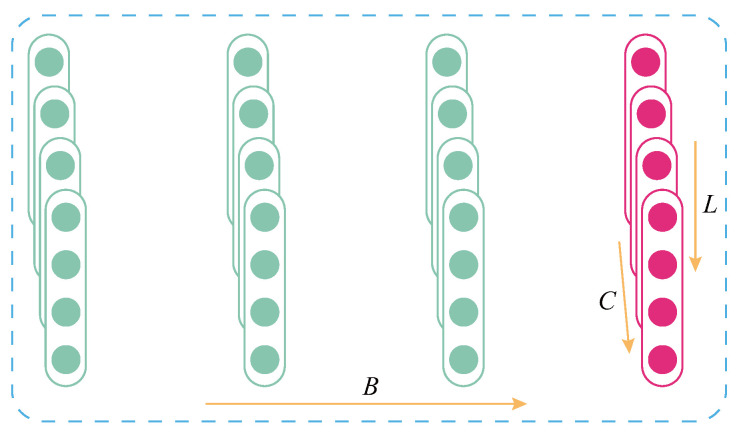
Visualization of layer normalization (normalized in the magenta part).

Based on the above parameter definitions, for any input feature map $x^{i}\in {ℜ}^{B\times C\times L}$ (where $x^{i}$ denotes the *i*-th input feature map, and ${ℜ}^{B\times C\times L}$ indicates that the feature map belongs to the real number space of dimension B×C×L), the mathematical expression of LN can be formulated as the following equation:

(5) $$\mathrm{LN}\left(x_{b,c,l}^{i}\right)=\frac{x_{b,c,l}^{i}-\mu^{i}}{\sqrt{{\left(\sigma^{i}\right)}^{2}+\varepsilon }}\gamma_{c,l}^{i}+\beta_{c,l}^{i}$$

where *b*, *c*, and *l* index the batch, channel, and feature, respectively; μ and σ are the mean and standard deviation (SD); ε is a small stabilizing constant; and γ and β are trainable scaling factors. As shown in Figure 2, LN computes μ and σ only over the channel (*C*) and feature (*L*) dimensions. Therefore, the mean and SD are calculated as follows:

(6) $$\mu^{i}=\frac{1}{CL}\sum_{c=1}^{C}\sum_{l=1}^{L}x_{b,c,l}^{i}$$

(7) $$\sigma^{i}=\sqrt{\frac{1}{CL}\sum_{c=1}^{C}\sum_{l=1}^{L}{\left(x_{b,c,l}^{i}-\mu^{i}\right)}^{2}}$$

### 2.3. Envelope Spectrum

Bearing fault signals typically exhibit distinctive modulation characteristics, primarily manifested as low-frequency periodic impacts (corresponding to fault characteristic frequencies) induced by localized damage. These impacts interact with the natural frequencies of the mechanical system, resulting in modulation into high-frequency resonance bands. This modulation phenomenon obscures the direct fault-related information within the complex frequency domain structure of the raw signal, making accurate identification of bearing faults a technical challenge. Demodulation analysis, serving as a core technique for processing such modulated signals, can effectively recover the low-frequency fault characteristic frequency components from the modulated signal, restoring them to their intrinsic frequency band positions in the spectrum. Consequently, demodulation analysis holds a fundamental position in the field of bearing fault diagnosis and has been extensively applied in both industrial practice and academic research [43].

Among various demodulation methods, envelope spectrum analysis stands out as a classical and mainstream tool due to its clear principles and relatively high computational efficiency. The core of this method lies in utilizing the Hilbert transform to extract the envelope of the vibration signal (i.e., the amplitude modulation information). Subsequently, the Fourier transform is applied to this envelope to obtain the signal’s envelope spectrum. The envelope spectrum highlights the periodic impact components within the signal, effectively achieving a “global” demodulation of the signal. This directly reveals the fault characteristic frequencies and their harmonics, making it highly favored by researchers, particularly for early fault diagnosis.

To elucidate the calculation process of the envelope spectrum, assume the original discrete vibration signal sequence to be analyzed is x[n], where n∈{0,1,…,N−1}, and *N* represents the total number of sampling points, i.e., the signal length. The envelope spectrum of this signal can be obtained through the following calculation steps:

(8) $$\begin{array}{c} \mathrm{ES}[m]=\frac{1}{N}\sum_{n=0}^{N-1}\tilde{x}\left[n\right]e^{-j2\pi nm/N},m\in \left\{0,1,\ldots ,N-1\right\} \end{array}$$

where $\tilde{x[n]}$ denotes the envelope of the signal x[n], given by the Hilbert transform as follows:

(9) $$\tilde{x}\left[n\right]=\sqrt{{\left(x\left[n\right]\right)}^{2}+{\left[H\left(x\left[n\right]\right)\right]}^{2}}$$

where H(·) is the Hilbert transform.

## 3. Methodology

### 3.1. Lightweight Network Module

Existing lightweight networks only use the number of network parameters as the measurement standard for lightweight models [30,31,32]. However, the number of parameters cannot reflect the model’s hardware requirements. Obviously, MACs and MF can better describe the hardware requirements of the model and are more suitable as criteria to measure lightweight models. Therefore, to achieve a lightweight network, then it is necessary to make the MACs and MF of the model as small as possible. The total MF of the model includes the MF of input, forward propagation, backward propagation, and parameters; so, the MF (M) of the model can be calculated as follows:

(10) $$\mathrm{MF}=\frac{4(I_{n}+F_{n}+B_{n}+P_{n})}{1024^{2}}$$

where $I_{n}$, $F_{n}$, $B_{n}$, $P_{n}$ represent the number of input, forward propagation, backward propagation, and parameters, respectively. For input feature maps $x\in {ℜ}^{B\times C\times L}$, $I_{n}$ can be expressed as follows:

(11) $$I_{n}=B\times C\times L$$

It can be seen from Equation (10) that the easier way to reduce MF is to reduce the number of input features $I_{n}$ and parameters $P_{n}$. To reduce the $I_{n}$, it is necessary to reduce the number of *B*, *C*, and *L* in the input feature maps x. *B* is a dynamic variable that can be dynamically adjusted according to hardware, and *C* is generally of 1, which means that to reduce $I_{n}$, it is only necessary to try to reduce *L* in x. From Equations (1)–(4), (10) and (11), it can be concluded that the network can be lightened by reducing *L* and adopting GC.

In recent years, modular deep learning methods has been widely used due to their simplicity of implementation [44]. This paper proposes an LNM, which mainly consists of a feature extraction layer, a normalization layer, and an activation layer. GC, LN, and ReLU are used as the feature extraction layer, the normalization method, and activation function of the LNM, respectively. Therefore, for input feature maps $x\in {ℜ}^{B\times C\times L}$, the forward propagation process of the jth LNM can be expressed as follows:

(12) $$x^{j+1}=\mathrm{ReLU}\left(\mathrm{LN}\left(\mathrm{GC}\left(x^{j}\right)\right)\right)$$

where ReLU activation function can be obtained as follows:

(13) ReLU(x)=max(0,x)

### 3.2. Dynamic Input Module

The ES, as a robust feature extraction method, is adopted as the input to the first LNM in this paper. However, directly feeding the full ES into the LNM would introduce substantial computational overhead, which is not an optimal design. Considering that the ES is a demodulated signal whose low-frequency region typically concentrates the most significant fault characteristic frequency components, the low-frequency segment of the ES can be truncated and used as the input to the LNM, thereby preserving critical information while reducing the computational load. Let the predefined input length of the first LNM be *L*; then, the first *L* sampling points of the ES need to be extracted as the input. It should be noted that the amount of information contained in these *L* sampling points is influenced by various factors such as sampling frequency and bearing operating conditions. Specifically, if *L* is too small, it will lead to insufficient frequency resolution and difficulty in effectively capturing fault characteristic frequencies; if *L* is too large, it will introduce redundant computation and degrade model efficiency. Therefore, this paper adopts a trade-off strategy: an adaptive length $L_{d}$ is selected to minimize computational cost while ensuring the required frequency resolution. Assume that the bandwidth corresponding to the first *L* sampling points of the truncated ES is denoted as *w*, which can be calculated as follows:

(14) $$w=\frac{Lf_{s}}{L_{d}}$$

where $f_{s}$ is the sampling frequency.

Generally, the bandwidth should exceed the bearing characteristic frequency. Considering that among the four typical bearing characteristic frequencies of rolling bearings, the ball pass frequency of the inner race is the highest fault frequency, and taking into account the need to capture up to three times the fault frequency components, *w* can be expressed as follows:

(15) $$w=3f_{i}$$

where $f_{i}$ denotes the ball pass frequency of the inner race, which can be calculated using the following formula:

(16) $$f_{i}=\frac{f_{r}}{2}z\left(1+\frac{d}{D}\cos\alpha \right)$$

where $f_{r}$, *d*, *D*, *z* and α are the rotating speed of the bearing, the rolling element diameter, the pitch circle diameter, the number of rolling elements and the contact angle, respectively. Combining Equations (14)–(16) to yield Equation (17), $L_{d}$ is dependent on parameters such as $f_{r}$, $f_{s}$, etc.

(17) $$L_{d}=\frac{2Lf_{s}}{3f_{r}z(1+\frac{d}{D}\cos\alpha )}$$

Based on Equation (17), the diagnostic length $L_{d}$ is positively correlated with *L* and $f_{s}$, while being inversely proportional to $f_{r}$ and the bearing structural parameter $b_{p}$. This relationship allows $L_{d}$ to adaptively adjust according to specific bearing configurations and sampling conditions. In other words, $L_{d}$ enables adaptive feature selection according to variations in bearing structural parameters, bearing rotational speed, and sampling frequency across different application scenarios. This ensures that no redundant computational cost is introduced while maintaining accuracy. To prioritize model efficiency (as analyzed in Section 3.1), we set L=400, which is significantly more compact than the conventional 2048 samples. Since the input dimension of the initial LNM is fixed, the variable $L_{d}$ cannot be directly processed. To bridge this gap, we propose the DIM, detailed in Algorithm 1, which maps the raw signal of length $L_{d}$ into a compatible input format for the LNM.
**Algorithm 1** The procedure of the DIM method1:  Acquire the raw vibration signal x[r] (r=1,2,…) via an acceleration sensor;2:  Compute the $L_{d}$ according to Equation (17);3:  Randomly sample a consecutive sequence of $L_{d}$ points from x[r] and denote it as $x[L_{d}]$;4:  Calculate the ES of $x[L_{d}]$ using Equation (8) and denote it as $\mathrm{ES}[L_{d}]$;5:  Extract the first *L* sample points from $\mathrm{ES}[L_{d}]$ and define the result as ES[d];6:  **Return** ES[d];

### 3.3. Lightweight Anti-Noise Diagnostic Framework

**Figure 3:**
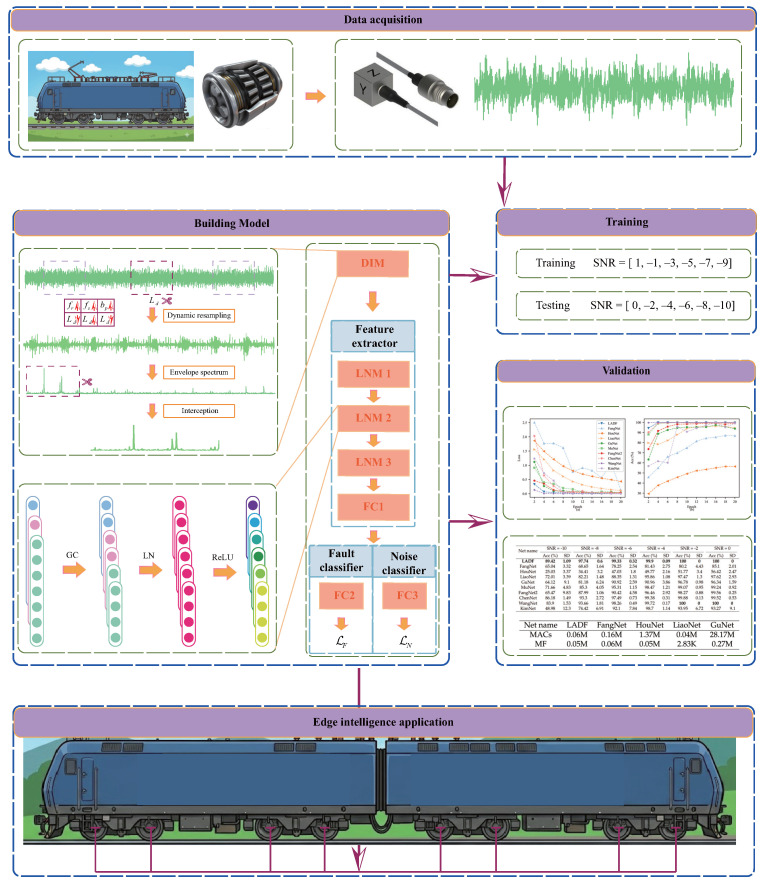
The flowchart of the proposed LADF.

This study proposes an LADF for train edge-end applications, designed to achieve accurate bearing fault diagnosis. The overall architecture of the framework is shown in Figure 3, and its structural parameters are detailed in Table 1. First, the DIM is employed to preprocess the data collected at the train edge-end. Subsequently, a feature extractor composed of multiple LNM modules is utilized for general feature extraction. Finally, the model is trained using an objective function that combines a fault classifier and a noise classifier. During training, noise with signal-to-noise ratios (SNR) of 1 dB, −1 dB, −3 dB, −5 dB, −7 dB, and −9 dB is injected into the collected data, thereby forcing the feature extractor to enhance its feature discriminative capability under noise interference. The objective functions of the fault classifier and the noise classifier are defined as follows:

(18) $$L_{C}=-\frac{1}{N}\sum_{i=1}^{N}\sum_{c=1}^{n_{c}}y_{ic}\log p_{ic}$$

(19) $$L_{N}=-\frac{1}{N}\sum_{i=1}^{N}\sum_{s=1}^{n_{s}}y_{is}\log p_{is}$$

where *N*, $n_{c}$ and $n_{s}$ are the number of samples, the number of fault types and the number of noise types, respectively. $y_{ic}$ denotes the true label of the *i*-th sample belonging to the *c*-th category. $p_{ic}$ denotes the predicted probability that the *i*-th sample is classified into the *c*-th fault category. Similarly, $y_{is}$ denotes the true label of the *i*-th sample corresponding to the *s*-th noise category. $p_{is}$ denotes the predicted probability that the *i*-th sample is classified into the *s*-th noise category. Integrating these objectives, the final optimization target is defined as follows:

(20) $$L=L_{C}+\lambda L_{N}$$

where λ represents the weighting factor for $L_{C}$ and $L_{N}$. Unlike static hyperparameters, λ is set to be trainable. Assuming $\theta_{f}$, $\theta_{c}$, and $\theta_{n}$ are the parameters of the feature extractor, fault classifier, and noise discriminator, respectively, the final optimization objective can be expressed as follows:

(21) $$\min_{\theta_{f},\theta_{c},\theta_{n},\lambda }\left[L_{C}\left(\theta_{f},\theta_{c}\right)+\lambda L_{n}\left(\theta_{f},\theta_{n}\right)+\ln\left(1+\frac{1}{\lambda^{2}}\right)\right]$$

The term $\ln(1+\frac{1}{\lambda^{2}})$ serves as a regularization term to prevent λ from converging to zero. The entire model can then be trained using the overall objective function (Equation (21)) and the Adam optimizer.

## 4. Experiments

This section systematically validates the robustness and fault identification efficacy of the LADF method under strong noise interference using two challenging RABB datasets. Among them, the RABB1 dataset simulates multiple operating conditions to test the model’s adaptability, while the RABB2 dataset is specifically designed for complex compound fault scenarios, further evaluating the method’s comprehensive diagnostic capability. Experimental results demonstrate that the proposed method remains stable and reliable even in noisy environments.

### 4.1. Experimental Setup

To comprehensively evaluate the proposed model’s integrated advantages in noise resistance capability and lightweight architecture, this study designs a detailed comparative experiment, systematically comparing it with nine state-of-the-art models. These nine models are divided into two categories: the first category comprises four lightweight models focused on computational efficiency, specifically including FangNet [32], HouNet [45], LiaoNet [46], and GuNet [47]; the second category consists of five noise-robust models specifically designed for noise resistance, namely MuNet [48], FangNet2 [49], ChenNet [50], WangNet [51], and KimNet [52]. All comparative models are strictly reproduced according to the structures and parameters described in their original papers to ensure the fairness of the comparison baseline.

To fully assess the stability and generalization capability of each model, all experiments are independently repeated 10 times, and all models maintain exactly the same structure and parameter configuration across both datasets (RABB1 and RABB2) to eliminate the influence of data-specific adaptability on the results. The experiments adopt uniform parameter settings to ensure fairness: the cross-entropy loss function is used; the Adam optimizer is employed with a dynamic learning rate schedule: $0.001/{\left(1+10\times i\right)}^{0.75}$, where parameter *i* linearly increases from 0 to 1 with the training process.; the number of training epochs, batch size, and the number of samples per fault category are set to 20, 128, and 300, respectively. The datasets are randomly divided into training, validation, and test sets in a 2:1:1 ratio. All models were implemented and evaluated under a unified experimental environment: Windows 10 operating system, PyTorch 2.0 framework, Intel Core i7-12700 CPU, and NVIDIA GeForce RTX 3070 Ti with 8GB GPU memory.

In terms of noise processing, to simulate extreme noise environments and enhance the models’ generalization ability, additive white Gaussian noise with SNR of 1 dB, −1 dB, −3 dB, −5 dB, −7 dB, and −9 dB is mixed into the training set during the training phase. During the model evaluation phase, the validation and test sets are evaluated using another set of SNR levels (0 dB, −2 dB, −4 dB, −6 dB, −8 dB, −10 dB). This approach aims to rigorously test the models’ robustness and noise resistance performance when facing unseen noise intensities.

### 4.2. Case 1: Bearing Fault Diagnosis Under Multiple Operating Conditions

#### 4.2.1. Introduction to RABB1 Dataset

**Figure 4:**
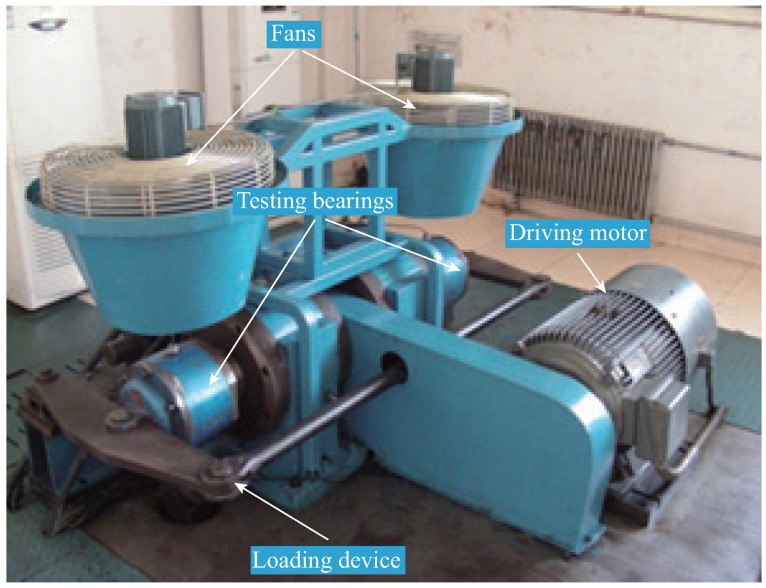
The test rig of the RABB1 dataset.

Under these circumstances, the dataset was collected from the RABB1 test rig, specifically designed to simulate multiple operating conditions, whose overall structure is shown in Figure 4 The core components of this test rig include a pair of test bearings, a driving motor, a loading device for applying vertical loads, and two fan motors for providing radial loads.

The key geometric parameters of the RABB1 test bearings are as follows: the number of rollers is 20, and the ball diameter (BD), pitch diameter (PD), and contact angle (CA) are 23.78 mm, 180 mm, and 0.174 rad, respectively. In the experiment, a total of one normal state and three typical single fault states are established, specifically including: normal state (N), rolling element fault (RF), inner raceway fault (IF), and outer raceway fault (OF). To comprehensively evaluate the performance of the diagnostic model under different operating conditions, data for each health state are collected under nine operating conditions formed by the combination of three different rotational speeds and three different vertical loads. Detailed information on the dataset composition is summarized in Table 2.

**Figure 5:**
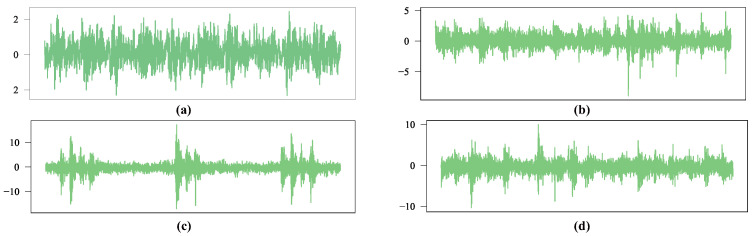
Raw time-domain waveforms of different health conditions in the RABB1 dataset. (**a**) N, (**b**) RF, (**c**) IF, (**d**) OF.

Vibration signal data are acquired using an accelerometer mounted on the bearing housing, with the sampling frequency set to 20 kHz. Figure 5 shows the raw vibration signal time-domain waveforms for these four health states (N, RF, IF, OF) under a typical operating condition, providing an intuitive observational basis for subsequent feature extraction and model analysis.

#### 4.2.2. Experimental Results

To comprehensively and thoroughly evaluate the overall performance and generalization capability of the proposed model, this section designs a systematic noise-testing environment and conducts detailed comparative experiments on this basis. Specifically, four recently published advanced lightweight models and five models specifically designed to enhance noise robustness are selected for the comparative experiments.

**Figure 6:**
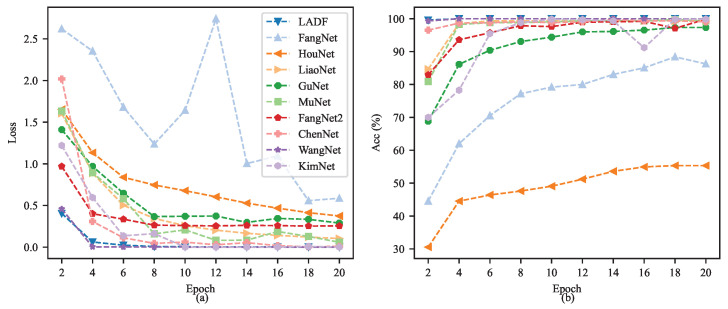
The learning curves (training loss (**a**) and validation accuracy (**b**)) of all evaluated models on the RABB1 dataset.

Figure 6 details a comparative analysis of the training process for ten different neural network models (including LADF, FangNet, HouNet, etc.) on the RABB1 dataset. The comparison employs a dual-metric evaluation over 20 epochs, presenting the training loss on the left (subfigure a) and the validation accuracy on the right (subfigure b). In the left loss curve, the majority of the models (e.g., LADF, WangNet, KimNet) demonstrate rapid convergence, with their loss values plummeting to near zero within the first few epochs. Notably, FangNet exhibits a significant loss rebound and fluctuation at the 12th epoch, while HouNet shows a consistently slow decline and ends with a relatively high final loss. Correspondingly, in the right accuracy curve, most models showcase excellent performance, swiftly achieving and maintaining validation accuracy close to 100%. In stark contrast, HouNet’s performance lags significantly, culminating in a final accuracy of merely approximately 55%. FangNet’s accuracy hovers around 85%. This visual representation underscores the overwhelming performance advantage and stability of models like LADF compared to HouNet on this specific task.

As indicated by the experimental results in Table 3, significant differences in classification accuracy (Acc) and standard deviation (SD) under various SNR conditions are observed among different methods on the RABB1 dataset. Overall, the LADF method consistently demonstrates the best performance across all SNR levels, with its superiority being particularly pronounced in low-SNR environments. For instance, at SNR = −10 dB, LADF achieves an accuracy of 90.31%, substantially outperforming other methods (e.g., FangNet: 65.49%, HouNet: 39.73%), while also exhibiting a very low standard deviation (1.05), indicating strong stability and robustness.

When SNR increases to 0 dB, LADF’s accuracy reaches approximately 99.7%, further confirming its adaptability under both high-noise and normal signal conditions. In contrast, HouNet and KimNet show notably poor performance at low SNR levels, reflecting their sensitivity to noise. While models such as FangNet, GuNet, and WangNet perform reasonably well at moderate to high SNRs, their accuracy declines significantly at −10 dB and −8 dB, and their stability is comparatively weaker. It is worth noting that ChenNet achieves an accuracy of 99.38% with a standard deviation of only 0.12 at SNR = 0 dB, demonstrating excellent precision and consistency. However, its performance deteriorates sharply under low-SNR conditions, indicating limited noise robustness. In summary, LADF not only maintains leading performance across all SNR settings but also exhibits strong generalization ability and stability, making it one of the most competitive robust methods for speech recognition or signal classification currently available. These results clearly demonstrate the superiority of LADF in complex noise environments and its suitability for real-world applications requiring high robustness.

**Figure 7:**
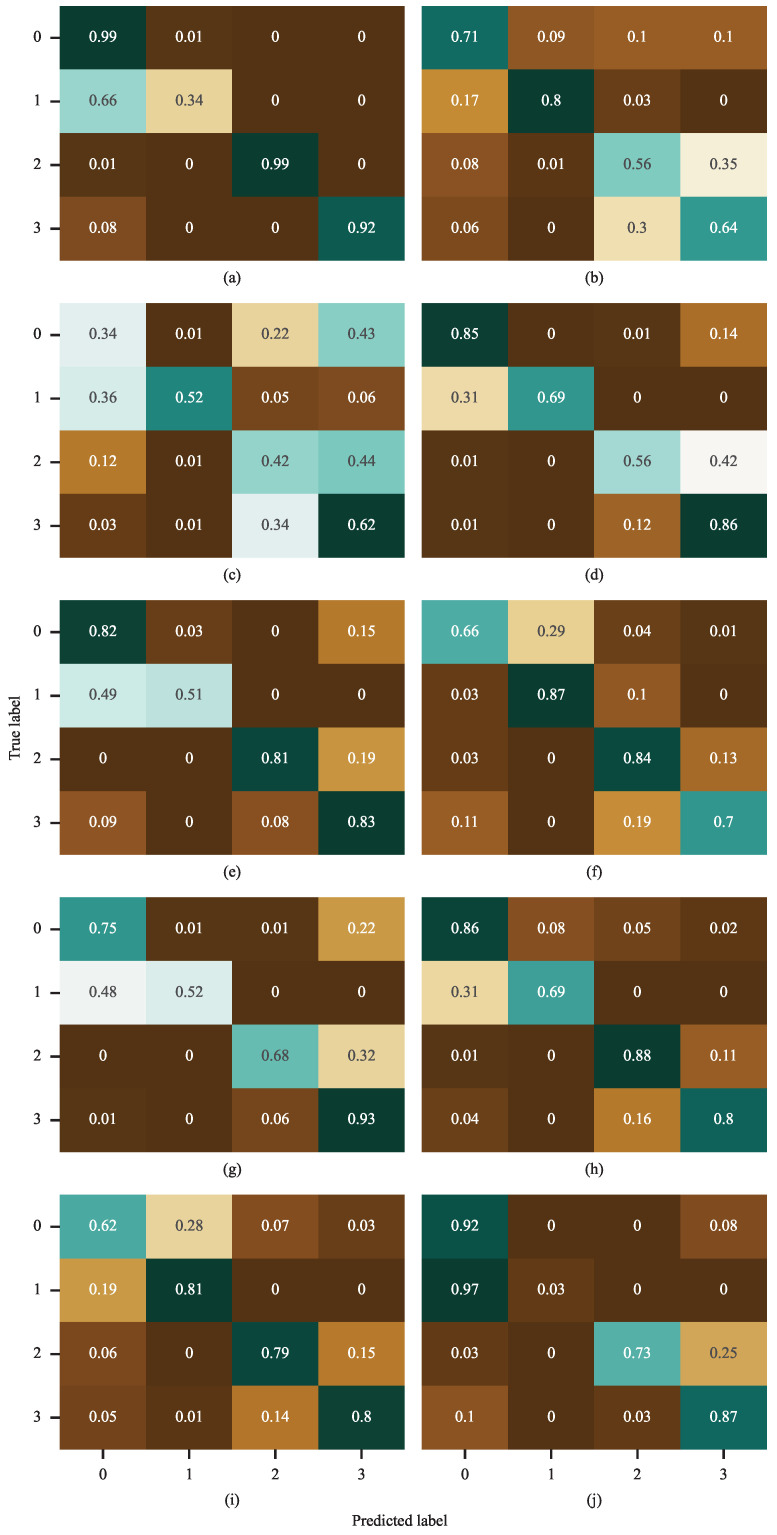
Confusion matrix of all models on the RABB1 dataset. (**a**) LADF, (**b**) FangNet, (**c**) HouNet, (**d**) LiaoNet, (**e**) GuNet, (**f**) MuNet, (**g**) FangNet2, (**h**) ChenNet, (**i**) WangNet, (**j**) KimNet.

Figure 7 visually presents the classification performance of ten different deep learning models on the RABB1 dataset under the highly challenging condition of an extremely low SNR = −10 dB, revealing significant differences among the models in terms of noise robustness and feature extraction capability. Specifically, LADF achieved prediction accuracy exceeding 90% for classes 0, 2, and 3. Its primary error occurred in class 1, where it incorrectly predicted class 1 as class 0 with a probability of 66%. Among the other comparative models, only FangNet2 surpassed 90% accuracy for class 3, and KimNet exceeded 90% accuracy for class 0. No other model achieved above 90% accuracy for any class. The performance of FangNet2 and KimNet on other classes was less satisfactory, with KimNet attaining an accuracy of only 3% for class 1. In conclusion, the proposed model, LADF, remains the best-performing model even under the most extreme experimental condition of −10 dB SNR, which validates its superior noise robustness.

### 4.3. Case 2: RABB Fault Diagnosis with Compound Faults

#### 4.3.1. Introduction to RABB2 Test Rig

**Figure 8:**
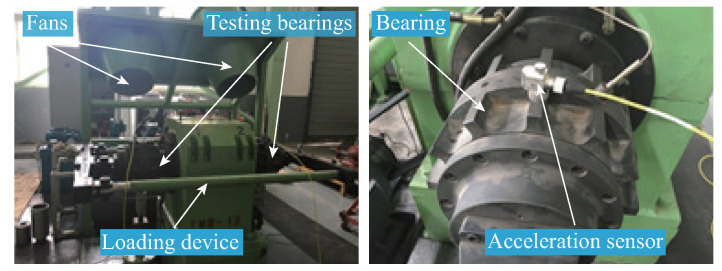
The test rig of the RABB2 dataset.

**Figure 9:**
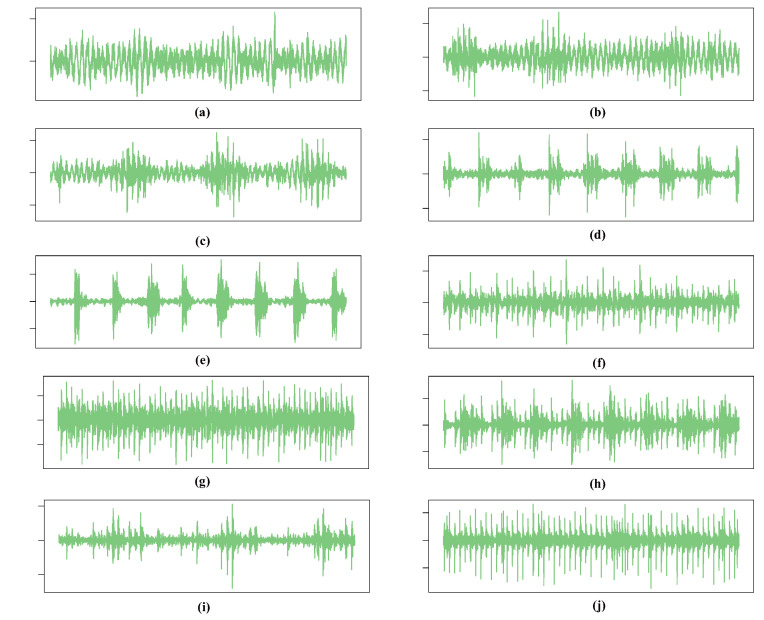
Raw time-domain waveforms of different health conditions in the RABB2 dataset. (**a**) N, (**b**) RF1, (**c**) RF2, (**d**) IF1, (**e**) IF2, (**f**) OF1, (**g**) OF2, (**h**) IOF, (**i**) ORF, (**j**) IORF.

To further validate the generalization capability of the proposed method, experiments were conducted using the RABB2 bearing dataset, which includes multiple fault types. The tested bearings were installed on the test rig shown in Figure 8, with the following structural parameters: number of rollers = 17, BD = 26.691 mm, PD = 187.205 mm, and CA = 0.211 rad. The dataset comprises one class of normal bearings and nine classes of artificially induced faulty bearings, specifically: Normal (N), Roller Fault with small damage size (RF1), Roller Fault with large damage size (RF2), Inner Race Fault with small damage size (IF1), Inner Race Fault with large damage size (IF2), Outer Race Fault with small damage size (OF1), Outer Race Fault with large damage size (OF2), combined Inner and Outer Race Fault (IOF), combined Outer Race and Roller Fault (ORF), and combined Inner Race, Outer Race, and Roller Fault (IORF). The experiments were performed under a wheelset speed of 100 km/h and a sampling frequency of 5120 Hz. The raw time-domain signals of the ten classes are shown in Figure 9, and the detailed configuration of the dataset is provided in Table 4.

#### 4.3.2. Experimental Results

Compared to high sampling frequency conditions, the use of a fixed input length at lower sampling frequencies enables coverage of a longer effective time window, allowing the model to capture more comprehensive fault evolution information and critical characteristics, thereby significantly enhancing diagnostic performance. However, as the number of fault categories increases and inter-class differences become more refined, complex and compound faults further reduce the inter-class separation, imposing higher demands on the model’s capability to extract discriminative features and delineate classification boundaries. To systematically evaluate the proposed model’s ability to recognize multi-category complex faults under low sampling frequency conditions, this section selects the RABB2 dataset, which includes multiple compound fault types, and conducts comparative experiments under various SNR scenarios. The proposed method is comprehensively compared against nine mainstream state-of-the-art models.

**Figure 10:**
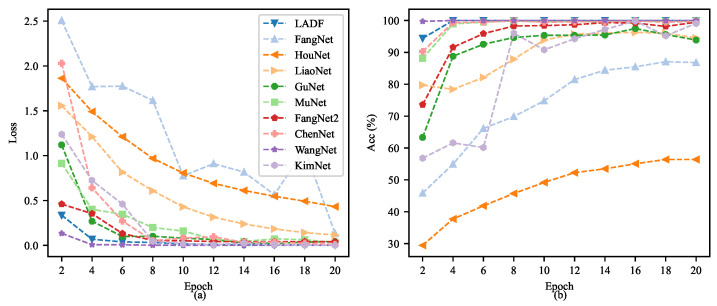
The learning curves (training loss (**a**) and validation accuracy (**b**)) of all evaluated models on the RABB2 dataset.

Based on the model training process curves on the RABB2 dataset depicted in Figure 10, we can conduct an in-depth, coherent and comprehensive analysis of the training dynamics of each model. In terms of the overall trend, distinct hierarchical disparities are exhibited by different models in terms of convergence speed and final performance, with the proposed LADF, WangNet and GuNet demonstrating superior learning capabilities. Specifically, the loss curves of these models plummeted to below 0.5 within the first 6 Epochs of training, then converged rapidly toward zero with extremely high stability—an indication that these network architectures can capture the key features of the data with exceptional sensitivity and complete parameter fitting efficiently. Such performance differences are more intuitively verified and amplified in the accuracy curves (Figure 10b). The top-performing model group, represented by LADF, saw its accuracy surge to nearly 100% at the very outset of training (approximately the 4th to 6th Epochs) and maintained an almost perfectly flat line in the subsequent training cycles. This not only attests to its ultra-high classification accuracy but also reflects its strong robustness, with no obvious overfitting-induced performance degradation observed. In summary, the experimental results strongly confirm that advanced models such as LADF hold overwhelming advantages over underperforming counterparts including HouNet in terms of feature extraction efficiency, convergence speed and final classification accuracy, thus validating their superior performance in practical applications.

**Figure 11:**
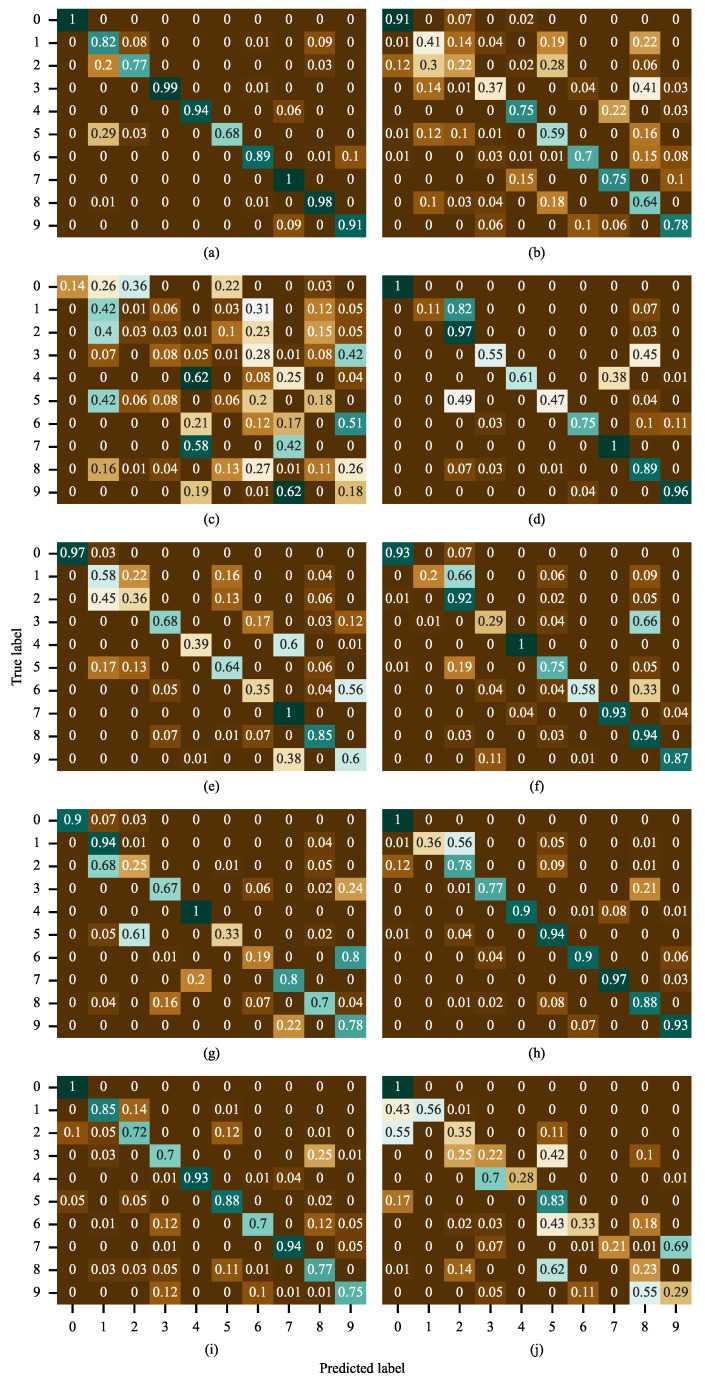
Confusion matrix of all models on the RABB2 dataset. (**a**) LADF, (**b**) FangNet, (**c**) HouNet, (**d**) LiaoNet, (**e**) GuNet, (**f**) MuNet, (**g**) FangNet2, (**h**) ChenNet, (**i**) WangNet, (**j**) KimNet.

Table 5 presents the experimental results of all models on the test set under different SNR ratios. With the increase in SNR from −10 dB to 0 dB, the performance of nearly all models shows a steady improvement, which is consistent with theoretical expectations. A higher SNR means less noise interference in the signal, making the effective features more prominent and thus easier for models to capture and identify. At the low SNR of −10 dB (a high-noise environment), the performance gap among the models is drastically widened: the state-of-the-art models can maintain an accuracy close to 90%, while the underperforming ones drop to approximately 25%. Even in the strongest noise environment at −10 dB, LADF still achieves a remarkable accuracy of 89.42% with a SD of merely 1.09, exhibiting extremely high performance stability. Notably, it is the only model that reaches an accuracy close to 90% at −10 dB across all tested models. When the SNR reaches −2 dB and 0 dB, LADF attains a perfect accuracy of 100% with an SD of 0, which indicates that it yields flawless predictions in every validation run under these conditions. These results further confirm LADF’s status as the optimal model in this experiment. It not only delivers perfect performance in the ideal environment at 0 dB but, more crucially, maintains an overwhelming advantage in the extreme high-noise environment at −10 dB—outperforming the second-ranked model by approximately 3.2% and the worst-performing one by around 64%. This fully demonstrates that the LADF algorithm has an extremely strong robustness against noise interference, rendering it highly suitable for practical application scenarios where signal interference is likely to exist. Figure 11 illustrates the confusion matrices of all models under an SNR of −10 dB. As can be observed from the figure, the proposed LADF model exhibits only low classification errors on Label 2 and Label 5. For all other labels, its classification accuracy exceeds 80%, and the accuracy of some labels even reaches 100%. Overall, LADF significantly outperforms all comparative models, which verifies that the proposed model possesses superior classification capability under extreme noise interference.

## 5. Discussion

### 5.1. The Comparison of Model Lightweight

To realize the deployment and application of the model in edge intelligence scenarios, it is necessary to significantly reduce its hardware resource consumption, with the core being to minimize the model’s multiply-accumulate operations (MACs) and memory footprint (MF) as much as possible. Table 6 compares the performance indicators of various lightweight models in terms of MACs, MF, and inference time (IT). As shown in the table, LiaoNet achieves the lowest computational overhead and the best lightweight performance, with a MAC value of only 0.04 M, which is 50% lower than that of the proposed LADF model (0.06 M). Meanwhile, its MF is merely 2.83 K and the inference time IT is only 1.02 ms, both of which are superior to the LADF model. The proposed LADF’s MACs, MF and IT are 0.06 M, 0.05 M, and 1.87 ms, respectively, ranking second in lightweight performance among all compared models. Although the LADF model is slightly inferior to LiaoNet in MACs, MF, and IT, the experimental results in Table 3 and Table 5 show that its classification accuracy is significantly higher than that of LiaoNet, especially in the strong noise environment (SNR = −10 dB), where the accuracy difference between the two exceeds 17%. This demonstrates that the proposed LADF model maintains high classification accuracy while still possessing excellent lightweight characteristics, achieving a well balance between model performance and edge deployment cost.

### 5.2. The Comparison of Batch Size

The selection of batch size exerts a crucial impact on the training speed of the model: a larger batch size accelerates the training process, yet it may give rise to out-of-memory (OOM) errors. For this reason, the performance of the proposed method under different batch sizes (BS) is investigated herein. The experimental results in Table 7 fully demonstrate that the proposed model exhibits excellent generalization ability and strong robustness across varying batch sizes. As the data shows, the model stably maintains a high diagnostic accuracy ranging from 89% to 91% on both the RABB1 and RABB2 datasets with an extremely small fluctuation (less than 1.5%), regardless of the input conditions of extreme single sample (BS = 1) or large batch (BS = 128). Notably, the model achieves optimal or near-optimal performance at BS = 1 (RABB1: 90.4%, RABB2: 90.87%). This not only verifies that the model adopts a normalization mechanism independent of batch statistical features (e.g., LN), thus overcoming the traditional challenge of unstable training with small batches, but also indicates that the model possesses extremely high flexibility for practical industrial deployment. It can adapt to various hardware environments, from edge devices with limited memory to high-performance computing centers, without sacrificing diagnostic accuracy by adjusting the batch size.

## 6. Ablation Experiments

### 6.1. Ablation Study of the DIM Module

To verify the necessity of DIM in the proposed LADF model and clarify how different input length (IL) configurations affect the model’s diagnostic performance, this study systematically evaluated the effects of various fixed IL and DIM settings on the performance of the LADF model, with the detailed experimental results presented in Table 8. All experiments were conducted under the same experimental setup (e.g., consistent optimizer parameters, training epochs, and data preprocessing procedures) to eliminate the interference of other factors, ensuring the reliability and comparability of the results. The experimental data clearly demonstrate a significant positive correlation between IL and model accuracy: as the IL increases within a reasonable range, the model can capture richer temporal features and more comprehensive fault-related information from the input signals, thereby markedly improving the diagnostic accuracy for fault identification. Specifically, a shorter IL may lead to the loss of key temporal details related to fault characteristics, resulting in insufficient feature extraction and lower diagnostic performance; in contrast, an appropriate increase in IL enables the model to capture the complete temporal evolution of fault signals, laying a solid foundation for accurate fault diagnosis.

In particular, when the DIM (proposed in this study) is adopted, the model achieves the optimal overall performance, with its accuracy reaching 90.31% and 89.42% on the RABB1 and RABB2 datasets, respectively. This outstanding performance is attributed to the inherent advantage of DIM: it can adaptively adjust the input length according to the actual fault characteristics of different datasets, rather than using a fixed IL that may be either too short or too long. This result indicates that DIM is capable of covering the complete fault cycle of the target signals, ensuring that the model extracts key fault features with sufficient discriminability and avoiding both the loss of critical information (caused by short fixed IL) and redundant computation (caused by overly long fixed IL).

Overall, under the DIM setting, the model stably achieves a high accuracy of approximately 90% on both the RABB1 and RABB2 datasets, exhibiting excellent consistency and robustness across different data distributions. This further confirms the necessity and effectiveness of introducing DIM into the LADF model, which not only improves the model’s diagnostic performance but also enhances its adaptability to different engineering application scenarios.

### 6.2. Ablation Study of the Normalization Methods

Normalization is a key technique in deep learning that accelerates model convergence and improves training efficiency by alleviating internal covariate shift, making it indispensable for network structures, especially fault diagnosis models. Different normalization methods yield varying performance improvements. To select the optimal one for the LADF model, we replace its original normalization with four mainstream methods: Batch Normalization (BN), Group Normalization (GN), Instance Normalization (IN), and LN, and conduct experiments under SNR of -10 dB. Specific experimental results are presented in Table 9. Results show LN exhibits superior anti-noise robustness, achieving the highest diagnostic accuracy of 90.31% and 89.42% on RABB1 and RABB2 datasets, respectively, due to its layer-wise normalization independent of batch size. BN and GN perform suboptimally, while IN is the worst with accuracy dropping to 56%, as it easily loses key fault features by focusing overly on individual instances. In summary, LN is the most effective and stable normalization strategy for the LADF model under harsh noise conditions.

## 7. Conclusions

In this paper, we proposed an LADF tailored for edge intelligence to address the challenges of deploying deep learning models on resource-constrained devices under harsh noise environments. By integrating three core modules, the LADF successfully overcomes the limitations of inflexible inputs, high computational costs, and poor noise immunity found in existing methods. Specifically, the DIM, based on the envelope spectrum, enables the framework to adaptively adjust to varying sampling frequencies and bearing parameters. The LNM, constructed with GC and LN, significantly reduces computational overhead while maintaining diagnostic precision. Furthermore, an auxiliary denoising branch is introduced to constrain the feature extractor, enhancing the model’s ability to learn generalized features under varying noise intensities. Extensive experiments on two railway axle box bearing datasets (RABB1 and RABB2) demonstrated the superiority of the proposed method. The results show that the LADF achieves a remarkable balance between performance and efficiency. Notably, it maintains a high diagnostic accuracy of approximately 90% even under extreme noise conditions of -10 dB, significantly outperforming state-of-the-art models. Additionally, the model exhibits exceptional stability across different batch sizes, validating its flexibility for diverse hardware deployments. In summary, the LADF provides a robust, efficient, and reliable solution for real-time bearing fault diagnosis in edge computing scenarios. 

## Figures and Tables

**Table 1:** The parameters of the proposed method.

Layer	Operations
LNM1	GC (*C* = 6, *K* = 1× 9, *G* = 1) → LN → ReLU → MP(*K* = 1×2)
LNM2	GC (*C* = 48, *K* = 1 × 9, *G* = 6) → LN → ReLU → MP(*K* = 1×2)
LNM3	GC (*C* = 48, *K* = 1 × 9, *G* = 48) → LN → ReLU → GAP
FC1	Linear(48,96) → ReLU → Linear(96,96) → ReLU
FC2	Linear(96,24) → ReLU → Linear(24,$n_{c}$) → Softmax
FC3	Linear(96,24) → ReLU → Linear(24,$n_{s}$) → Softmax

Note: *C* denotes the number of channels, *K* represents the kernel size, and *G* is the number of groups in GC. MP and GAP refer to max pooling and global average pooling, respectively. Additionally, $n_{c}$ and $n_{s}$ correspond to the number of fault categories and noise categories, respectively.

**Table 2:** Details of the RABB1 datasets.

Samples	Fault Type	Label	Speed (km/h)	Load (kN)	Damage Size (mm)
300	N	0	90/120/150	56/146/236	-
300	RF	1	90/120/150	56/146/236	3×45
300	IF	2	90/120/150	56/146/236	3×45
300	OF	3	90/120/150	56/146/236	10×30

**Table 3:** Experimental results of all methods on the RABB1 dataset under different SNRs.

Net Name	SNR = −10		SNR = −8		SNR = −6		SNR = −4		SNR = −2		SNR = 0	
	Acc (%)	SD	Acc (%)	SD	Acc (%)	SD	Acc (%)	SD	Acc (%)	SD	Acc (%)	SD
**LADF**	**90.31**	**1.05**	**98.73**	**0.81**	**99.69**	**0.23**	**99.6**	**0.24**	**99.91**	**0.12**	**99.7**	0.33
FangNet	65.49	2.64	72.02	3.89	75.7	4.34	81.92	6.3	84.01	2.23	88.47	2.7
HouNet	39.73	4.6	54.63	2.36	57.03	2.62	59.7	3.26	61.7	2.22	62.74	3.27
LiaoNet	76.82	3.12	88.2	3.69	94.42	1.03	97.7	1.2	97.45	2.76	99.26	0.43
GuNet	74.1	2.31	81.4	1.62	88.89	2.44	93.32	0.89	93.8	1.78	95.42	1.21
MuNet	76.9	5.69	88.7	3.03	88.34	11.2	97.42	0.87	88.23	10.4	73.6	14.64
FangNet2	74.9	3.14	82.88	2.92	86.1	3.43	90.62	4.24	94.02	4.37	94.3	2.15
ChenNet	78.1	3.22	86.68	3	95.03	0.96	96.02	1.6	98.11	1.02	99.38	**0.12**
WangNet	77.13	1.8	82.22	4.52	92.81	1.9	96.82	1.19	98.42	0.8	98.26	1.13
KimNet	61.9	8.6	80.74	4.93	93.75	1.59	97.05	1.62	94.9	3.43	96.05	1.9

**Table 4:** Details of the RABB2 datasets.

Samples	Fault Type	Label	Speed (km/h)	Damage Size (mm)
300	N	0	100	-
300	RF1	1	100	0.2×0.3
300	RF2	2	100	0.2×0.6
300	IF1	3	100	0.2×0.3
300	IF2	4	100	0.2×0.6
300	OF1	5	100	0.2×0.3
300	OF2	6	100	0.2×0.6
300	IOF	7	100	0.2×0.6
300	ORF	8	100	0.2×0.6
300	IORF	9	100	0.2×0.6

**Table 5:** Detailed test information for all methods at different SNRs on RABB2 datasets.

Net Name	SNR = −10		SNR = −8		SNR = −6		SNR = −4		SNR = −2		SNR = 0	
	Acc (%)	SD	Acc (%)	SD	Acc (%)	SD	Acc (%)	SD	Acc (%)	SD	Acc (%)	SD
**LADF**	**89.42**	**1.09**	**97.74**	**0.6**	**99.33**	**0.32**	**99.9**	**0.09**	**100**	**0**	**100**	**0**
FangNet	65.04	3.32	68.65	1.64	78.25	2.54	81.43	2.75	80.2	4.43	85.1	2.01
HouNet	25.03	3.37	34.41	3.2	47.03	1.8	49.77	2.16	51.77	3.4	56.42	2.47
LiaoNet	72.01	3.39	82.21	1.48	88.35	1.31	95.86	1.08	97.47	1.3	97.62	2.93
GuNet	64.12	9.1	81.18	6.24	90.92	2.59	90.96	3.86	96.78	0.98	96.34	1.59
MuNet	71.66	4.83	85.3	4.05	95.31	1.15	98.47	1.21	99.07	0.95	99.24	0.92
FangNet2	65.47	9.83	87.99	1.06	90.42	4.58	96.46	2.92	98.27	0.88	99.56	0.25
ChenNet	86.18	1.49	93.3	2.72	97.49	0.73	99.38	0.31	99.88	0.13	99.52	0.53
WangNet	83.9	1.53	93.66	1.81	98.26	0.49	99.72	0.17	**100**	**0**	**100**	**0**
KimNet	48.98	12.3	74.42	6.91	92.1	7.84	98.7	1.14	93.95	6.72	93.27	9.1

**Table 6:** Comparision of MACs, MF and IF for lightweight models.

Net Name	LADF	FangNet	HouNet	LiaoNet	GuNet
MACs	0.06 M	0.16 M	1.37 M	0.04 M	28.17 M
MF	0.05 M	0.06 M	0.05 M	2.83 K	0.27 M
IT	1.87 ms	2.48 ms	14.49 ms	1.02 ms	71.32 ms

**Table 7:** Generalization ability of the model under different batch sizes.

Dataset	BS = 1	BS = 16	BS = 64	BS = 128
RABB1	90.4	90.33	89.0	90.31
RABB2	90.87	90.13	89.67	89.42

**Table 8:** Model accuracy (%) under different IL.

Dataset	IL = 1024	IL = 2048	IL = 4096	IL = DIM
RABB1	43.39	46.82	62.32	90.31
RABB2	43.2	56.56	77.48	89.42

**Table 9:** Diagnostic accuracy of the LADF under different normalization methods.

Dataset	BN	GN	IN	LN
RABB1	87.07	85.67	56.87	90.31
RABB2	83.47	82.67	55.2	89.42

## Data Availability

The DAS in this paper is unavailable for confidentiality reasons.
